# Genotoxicity assessment: opportunities, challenges and perspectives for quantitative evaluations of dose–response data

**DOI:** 10.1007/s00204-023-03553-w

**Published:** 2023-07-05

**Authors:** Jakob Menz, Mario E. Götz, Ulrike Gündel, Rainer Gürtler, Kristin Herrmann, Stefanie Hessel-Pras, Carsten Kneuer, Franziska Kolrep, Dana Nitzsche, Ulrike Pabel, Benjamin Sachse, Sebastian Schmeisser, David M. Schumacher, Tanja Schwerdtle, Tewes Tralau, Sebastian Zellmer, Bernd Schäfer

**Affiliations:** 1 Department of Food Safety, German Federal Institute for Risk Assessment (BfR), Max-Dohrn-Str. 8-10, 10589 Berlin, Germany; 2 Department of Chemical and Product Safety, German Federal Institute for Risk Assessment (BfR), Max-Dohrn-Str. 8-10, 10589 Berlin, Germany; 3 Department of Pesticides Safety, German Federal Institute for Risk Assessment (BfR), Max-Dohrn-Str. 8-10, 10589 Berlin, Germany; 4 Department of Safety in the Food Chain, German Federal Institute for Risk Assessment (BfR), Max-Dohrn-Str. 8-10, 10589 Berlin, Germany; 5 German Federal Institute for Risk Assessment (BfR), Max-Dohrn-Str. 8-10, 10589 Berlin, Germany

**Keywords:** Genetic toxicology, Dose–response analysis, Point of departure, Margin of exposure, Health-based guidance value, Risk assessment

## Abstract

Genotoxicity data are mainly interpreted in a qualitative way, which typically results in a binary classification of chemical entities. For more than a decade, there has been a discussion about the need for a paradigm shift in this regard. Here, we review current opportunities, challenges and perspectives for a more quantitative approach to genotoxicity assessment. Currently discussed opportunities mainly include the determination of a reference point (e.g., a benchmark dose) from genetic toxicity dose–response data, followed by calculation of a margin of exposure (MOE) or derivation of a health-based guidance value (HBGV). In addition to new opportunities, major challenges emerge with the quantitative interpretation of genotoxicity data. These are mainly rooted in the limited capability of standard in vivo genotoxicity testing methods to detect different types of genetic damage in multiple target tissues and the unknown quantitative relationships between measurable genotoxic effects and the probability of experiencing an adverse health outcome. In addition, with respect to DNA-reactive mutagens, the question arises whether the widely accepted assumption of a non-threshold dose–response relationship is at all compatible with the derivation of a HBGV. Therefore, at present, any quantitative genotoxicity assessment approach remains to be evaluated case-by-case. The quantitative interpretation of in vivo genotoxicity data for prioritization purposes, e.g., in connection with the MOE approach, could be seen as a promising opportunity for routine application. However, additional research is needed to assess whether it is possible to define a genotoxicity-derived MOE that can be considered indicative of a low level of concern. To further advance quantitative genotoxicity assessment, priority should be given to the development of new experimental methods to provide a deeper mechanistic understanding and a more comprehensive basis for the analysis of dose–response relationships.

**Supplementary Information:**

The online version contains supplementary material available at 10.1007/s00204-023-03553-w.

## Introduction

Genotoxicity testing results take a special role in the assessment and management of chemical risks to consumers. This is mainly due to the irreversible nature and the particular severity of the adverse health effects that may result from genotoxic events. According to the Globally Harmonized System of Classification and Labeling of Chemicals (GHS), the general terms ‘genotoxic’ and ‘genotoxicity’ apply to “agents or processes which alter the structure, information content, or segregation of DNA, including those which cause DNA damage by interfering with normal replication processes, or which in a non-physiological manner (temporarily) alter its replication” (UN 2021). In addition, the more specific terms ‘mutagenic’ and ‘mutagen’ are used for agents “giving rise to an increased occurrence of mutations in populations of cells and/or organisms”, with the term ‘mutation’ referring to “a permanent change in the amount or structure of the genetic material in a cell” (UN 2021).

Genotoxic substances alter the structure, information content, or segregation of DNA in different ways. ‘DNA-reactive’ substances primarily cause DNA damage by forming covalent DNA adducts and by cross-linking (i.e., intrastrand DNA cross-links, interstrand DNA cross-links, and DNA–protein cross-links). In contrast, ‘non-DNA-reactive’ genotoxic substances give rise to DNA damage through indirect mechanisms, for example by the generation of reactive oxygen species (ROS), or by interfering with cellular components involved in maintaining genomic stability, chromosome integrity, or functioning of the mitotic spindle (WHO/FAO 2020a). In replicating cells, directly or indirectly induced DNA damage, if not repaired in time, can ultimately lead to different types of mutations. These are broadly grouped into gene mutations (i.e., changes in single DNA bases and small intragenic insertions/deletions and rearrangements), structural chromosomal aberrations (i.e., chromosome breaks and rearrangements; clastogenicity), and numerical chromosomal aberrations (i.e., gain or loss of a whole chromosome; aneugenicity) (EFSA 2021; Zeiger 2010a). With regard to gene mutations, it is worth noting that the very same types of mutations also occur in non-coding intergenic regions of the DNA.

Mutations also arise spontaneously in the genome of an organism. In this context, it is worth noting that the frequency of random spontaneous mutations is not a constant term but differs between organisms, tissues and even genomic locations. For this reason, a substance-induced increase in the mutation frequency can only be determined in relation to the corresponding endogenous background. This endogenous background is the result of a complex interplay of various processes that, on the one hand, induce DNA damage and mutations and, on the other hand, maintain genetic stability. For instance, base mismatches during DNA replication are considered a major cause of spontaneous mutations. In eukaryotic cells, DNA polymerization through major replicative DNA polymerases (α, δ, and ε) generates around one mismatch per every 10^4^ to 10^5^ bases incorporated (Kunkel and Erie 2015). However, under normal circumstances, most base mismatches are effectively corrected by the proofreading exonuclease activity of replicative DNA polymerases and to a lesser extent also by postreplicative DNA mismatch repair (Kunkel and Erie 2015). Beyond that, other endogenous processes such as the formation of DNA-reactive metabolites (e.g., during physiologic metabolism of carbohydrates, lipids and amino acids), generation of ROS, hydrolytic cleavage of purine bases (depurination), and hydrolytic conversion of cytosine to uracil (deamination) give rise to spontaneous DNA lesions. These in turn are tightly monitored and generally effectively repaired in healthy individuals by a complex network of lesion-specific DNA damage response mechanisms (Chatterjee and Walker 2017; Jackson and Bartek 2009). The high fidelity of DNA replication and repair is also reflected by the exceedingly low mutation frequencies in human genomes. As an illustration, the frequency of random single-nucleotide substitutions in normal (non-neoplastic) human tissues has been estimated to be less than 1 × 10^−8^ per base pair (Bielas et al. 2006).

Whether a mutation results in an adverse health outcome and what type of outcome is expressed at the phenotypic level depends very much on the particular genetic background, the type of mutation and the particular genetic locus, cell type, tissue and life stage affected. In germ cells, a single mutation in a single cell may adversely affect the offspring and subsequent generations, because, in the event of fertilization, it may be passed on to every somatic and germline cell of the progeny. For this reason, and also due to possible direct effects on reproduction, such as infertility, spontaneous abortions, and congenital malformations, the identification of germ cell mutagens is considered a particularly important task (EFSA 2011; OECD 2017). Also in somatic cells, some mutations are clearly associated with a higher risk of developing a disease. This applies in particular to mutations in so-called ‘cancer driver genes’, which account for about 200 of the 20,000 genes in the human genome (Vogelstein and Kinzler 2015). In most cases, at least three mutations in a particular subset of driver genes are required for a normal cell to progress to an advanced cancer (Tomasetti et al. 2015; Vogelstein and Kinzler 2015). However, there are also examples, such as in chronic myeloid leukemia, where a single “hit” can be sufficient to convert a normal stem cell into a tumor cell (Melo and Barnes 2007). In addition, the acquisition of somatic mutations at the postzygotic developmental stage and subsequent clonal expansion of mutated cells leads to a phenomenon termed ‘genetic mosaicism’ (Godschalk et al. 2020; Martinez-Glez et al. 2020). There is growing evidence of a contribution of genetic mosaicism to various monogenic and complex diseases (Godschalk et al. 2020). Out of the many known variants, some genetic mosaics have been associated with severe health outcomes such as miscarriage, congenital anomalies, developmental delay, and cancer (Martinez-Glez et al. 2020).

The high sensitivity of modern analytical techniques creates a situation in which an increasing number of natural as well as synthetic genotoxicants can be detected in minute amounts, be it in the environment, in food, in consumer goods, or in pharmaceutical products. Although targeted analytical studies usually focus on specific analytes in selected matrices, the totality of available occurrence data suggests that the general population is continuously exposed to a variety of genotoxic agents from many different sources (Wogan et al. 2004).

Genotoxic substances not intentionally used but present for other reasons, e.g., due to natural occurrence in raw materials or contamination during manufacture or processing of products, are often regulated according to the principle of reducing consumer exposure to a level *as low as reasonably achievable* (ALARA). The rationale behind such regulations roots in the so-called “one-hit” or “single-hit” hypothesis, according to which exposure to even a single genotoxic molecule could trigger a harmful mutation and thus increase the risk to develop a genetic disease (EFSA 2005; WHO/FAO 2020a). Other relevant aspects supporting the implementation of the ALARA principle are the severity, irreversibility and delayed manifestation of possible health effects as well as the inherent difficulties in assessing combined effects that may result from co-exposure and aggregated exposure to multiple genotoxic substances from multiple sources.

The intentional use of genotoxic chemicals is tightly regulated with restrictions applying for many areas of application. For instance, in the European Union (EU), substances with a harmonized classification for germ cell mutagenicity (hazard categories Muta. 1A and Muta. 1B) fall under Entry 29 of Annex XVII of Regulation (EU) No 1907/2006 (REACH), which results in a ban of the supply of those substances (as such or in mixtures) to the general public. In addition, many substance- or product-centered regulations (e.g., for active substances in pesticides, biocides or for cosmetic ingredients) include hazard-based exclusion criteria for known mutagens (Muta. 1A and 1B) and generic risk management obligations for suspected germ cell mutagens (Muta. 2).

The mainly hazard-oriented approach for regulating genotoxic substances creates a situation in which regulatory decisions depend to a considerable extent on a purely qualitative and binary (“yes or no”) interpretation of genotoxicity data. Over the last decade, several authors have criticized this assessment paradigm for only considering genotoxic activity as such, not taking into account any differences in genotoxic potency (Gollapudi et al. 2013; Johnson et al. 2014; MacGregor et al. 2015a, 2015b; White and Johnson 2016; White et al. 2020). This has prompted a multifaceted discussion about the need of a paradigm shift towards a stronger consideration of genetic toxicity dose–response data. In light of these recent developments, the objectives of this review are (i) to provide a short introduction to the traditional paradigm of genotoxicity assessment, (ii) to briefly review currently discussed opportunities involving quantitative interpretation of genotoxicity data, (iii) to identify new challenges arising from quantitative interpretation of genotoxicity data, and (iv) to discuss the perspectives for the integration of quantitative approaches to genotoxicity assessment.

## The traditional paradigm of genotoxicity assessment

### Hazard identification

In chemical risk assessment, hazard identification is defined as “the identification of the type and nature of adverse effects that an agent has as inherent capacity to cause in an organism, system, or (sub)population” (OECD 2004). Starting from the late 1960s, the assessment of genotoxic effects of chemicals has received increasing attention and became an essential part of the hazard identification step. This growing interest in identifying genotoxic chemicals has spawned a plethora of in vivo*, *in vitro and in silico methods for the detection or prediction of a wide range of genetic toxicity endpoints (Mahadevan et al. 2011; Zeiger 2010b). Here, we focus on a short overview of some particularly important test methods and evaluation principles. For a more comprehensive overview on current approaches to the identification of genotoxic properties of chemicals, reference is made to the detailed guidance documents and scientific opinions of the international risk assessment bodies (e.g., ECHA 2017; EFSA 2011; EFSA 2017; OECD 2017; WHO/FAO 2020a).

The publication of the Salmonella reverse mutation assay (“Ames test”) by Bruce Ames and colleagues in 1973 (Ames et al. 1973) marked an unprecedented innovation in the field of genotoxicity testing. This in vitro method, also known as the bacterial reverse mutation test (OECD TG 471), has greatly contributed to the advancement of genotoxicity testing and takes a prominent role in regulatory assessment schemes until today (Zeiger 2019). In addition, mammalian cell culture based methods, such as gene mutation tests using the thymidine kinase (*tk*) gene (OECD TG 490) or the *Hprt* and *xprt* genes (OECD TG 476) as reporter genes, the chromosomal aberration test (OECD TG 473), and the micronucleus test (OECD TG 487) have become important cornerstones of current in vitro genotoxicity testing batteries. The combinations of in vitro tests typically used in regulatory assessments show a high sensitivity with regard to the identification of in vivo genotoxicants and rodent carcinogens (Kirkland et al. 2011). However, at the same time, a rather low specificity, i.e. the ability to correctly predict negative results in vivo, has been reported (Ates et al. 2014; Kirkland et al. 2007).

Many regulatory frameworks also require in vivo genotoxicity studies, either per se or as follow-up to positive in vitro results (EC 2013; ECHA 2017; ECHA 2022; EFSA 2011). Depending on the genotoxicity endpoint tested positive in vitro, a transgenic rodent gene mutation assay (OECD TG 488), an in vivo mammalian alkaline comet assay (OECD TG 489) and/or a mammalian erythrocyte micronucleus test (OECD TG 474) or a mammalian bone marrow chromosomal aberration test (OECD TG 475) are usually considered as appropriate follow-up investigations (ECHA 2017; EFSA 2011). The mammalian erythrocyte *Pig-a* gene mutation assay, for which an OECD test guideline (OECD TG 470) has recently been published, is expected to be used as a follow-up method in the near future (OECD 2020a, b). In addition, there are certain in vivo methods such as the rodent dominant lethal mutation test (OECD 478) or the mouse heritable translocation assay (OECD 485), which are specifically designed to detect heritable germ cell mutations.

The identification of genotoxic effects is increasingly supported by an initial in silico screening phase, in particular when the compound under assessment is not readily available for experimental testing or a prioritization from a larger set of substances has to be achieved. In addition, in silico methods have the potential to identify the structural features of a mutagenic compound related to an observed mutagenic effect and thus provide some mechanistic information complementing the in vitro and in vivo studies discussed above.

The hazard identification step typically concludes with a thorough review of the entire genotoxicity data package using a weight of evidence approach. In this context, direct or indirect evidence supporting exposure of the analyzed target tissues (i.e., the bone marrow in case of OECD TG 474 or OECD TG 475) is usually mandatory to consider an in vivo study as (clearly) negative (EFSA 2017). Moreover, positive results from test methods based on measurements of primary DNA damage, such as the in vivo mammalian alkaline comet assay (OECD TG 489) and the in vivo unscheduled DNA synthesis (UDS) test (OECD TG 486), are usually interpreted only as indicators of mutagenic effects. If all available information taken together provides evidence of a mutagenic effect that is also expressed in vivo*,* this is usually regarded as an indication for a potential carcinogenic hazard. Moreover, if there is evidence for mutagenic effects in germ cells, the substance might also be considered for classification as a (possible) germ cell mutagen (UN 2021).

### Hazard characterization

The Organisation for Economic Co-operation and Development (OECD) defines hazard characterization as follows: “The qualitative and, wherever possible, quantitative description of the inherent property of an agent or situation having the potential to cause adverse effects. This should, where possible, include a dose–response assessment and its attendant uncertainties” (OECD 2004)*.* As already mentioned, regulation of genotoxic chemicals is mainly hazard-oriented with restrictions applying for most areas of application. For use categories falling under such a restriction, there is usually no need to proceed with the risk assessment if the hazard identification step provided clear evidence of genotoxicity. However, there are situations, for example in the event of unintended or unavoidable exposure of consumers, that still require a dose–response assessment and the estimation of a low-effect level. This low-effect level is usually referred to as the ‘reference point’ or ‘point of departure’ (PoD).^1^

Under the traditional assessment paradigm, dose–response analysis and reference point determination mainly focus on downstream apical effects. However, the use of apical endpoint data for assessing dose–response relationships of genotoxic substances has inherent limitations. Many genotoxic substances are believed to induce stochastic effects. This means that the occurrence of effects is random with the probability of occurrence depending on the dose absorbed—or in other words, the chance of experiencing the effect increases with increasing dose (Herber et al. 2001). The recognition of an adverse outcome as the result of a stochastic process has crucial implications for the interpretation of apical endpoint data. Given that only a limited number of animals can be included in a toxicological study, adverse events that occur only sporadically in the studied population will remain largely unobserved. Thus, for genotoxic compounds, severe adverse effects must be expected to go undetected below a certain dose/incidence level. For example, in a long-term carcinogenicity study typically involving 50 animals of each sex per dose group, the smallest detectable significant increase above the non-exposed control is often close to 10% (EFSA 2005; Sand et al. 2011; US EPA 2012). Therefore, carcinogenicity testing is usually performed at high doses up to the maximum tolerated dose (MTD) in order to minimize the risk of false negative results (OECD 2014). However, this strategy might result in an overestimation of the carcinogenic risk with respect to the human situation because of the saturation of detoxification and repair mechanisms and the activation of secondary toxicity pathways (Felter et al. 2020; Goodman 2018; Hartwig et al. 2020).

In the specific context of carcinogenicity test results, different methods have been applied to characterize dose–response relationships and/or to determine a toxicological reference point. These include the no-observed-adverse-effect level (NOAEL) approach, the T25^2^ approach, the TD_50_^3^ approach, and the benchmark dose (BMD) approach (Crump 2018; EFSA 2005). The BMD approach, which is based on the fitting of a mathematical model (or group of models) to the experimental dose–response data, is considered as the preferred method for both cancer and non-cancer endpoints (EFSA 2005; EFSA 2022; US EPA 2012). If the BMD approach is applied to a carcinogenicity study, the lower bound of the estimated dose corresponding to a response of a 10% extra tumor incidence (i.e., the BMDL_10_) is typically used as the toxicological reference point (EFSA 2005). Moreover, in cases where the available dose–response data is not suitable for deriving a meaningful BMDL_10_, the use of alternative potency metrics such as the BMD_10_ or T25 is also considered an option (ECHA 2017; EFSA 2005).

Specific rules apply to the determination of toxicological reference points for genotoxic substances that are considered purely aneugenic. EFSA recommends that “reference points for aneugenicity should be identified by analysis of dose–response data for induction of micronuclei from in vivo studies” (EFSA 2021). Likewise, the European Chemicals Agency (ECHA) has pointed out that, on certain occasions, e.g., when the effect is caused by aneugenic mechanisms, determination of dose–response relationships in genotoxicity assays may be a helpful tool in risk assessment (ECHA 2017). However, it is important to note that purely aneugenic substances are generally assumed to have non-DNA-reactive mechanisms responsible for their genotoxic effects. Thus, in this particular case, estimating a threshold dose from appropriate in vivo data seems possible. In this context, EFSA recommends that toxicological endpoints other than genotoxicity may be included in the dose–response assessment to identify the most sensitive effect (EFSA 2021).

### Risk characterization

In order to characterize the risk associated with consumer exposure to substances that are genotoxic and carcinogenic, risk assessors in the EU usually apply either the margin of exposure (MOE) concept according to EFSA (2005) or the derived minimal effect level (DMEL) approach according to ECHA (2012). The MOE is the ratio between a toxicological reference point from an adequate toxicity or carcinogenicity study and the estimated human exposure. According to EFSA, a MOE of 10,000 or higher that is based on a BMDL_10_ from a carcinogenicity study in rodents “would be of low concern from a public health point of view and might be considered as a low priority for risk management actions” (EFSA 2005). Thus, the MOE approach is intended to provide a rough classification of the level of concern that is associated with a given exposure. The DMEL approach for non-threshold carcinogens differs in this respect, as it aims to derive “an exposure level corresponding to a low, possibly theoretical, risk” (ECHA 2012). Like the MOE approach, the DMEL approach uses a reference point from a carcinogenicity study. Starting from this reference point, the DMEL is calculated either by linear high- to low-dose extrapolation to a specific risk level (“linearized approach”) or by applying multiple assessment factors (AF) (“large assessment factor approach”). With respect to the linearized approach, ECHA suggests that a theoretical cancer risk of 10^–6^ could be seen as indicative tolerable risk level for establishing a DMEL for the general population (ECHA 2012). In connection with the large assessment factor approach, a default overall AF of 10,000 is recommended to derive a DMEL for the general population based on a BMDL_10_ (ECHA 2012).

A major drawback of the current application of both the MOE and the DMEL approach is the restriction to substances for which appropriate carcinogenicity data are available. Carcinogenicity studies are generally time-consuming, expensive, require large numbers of animals and are often not part of the regulatory standard information requirements. This creates a situation where the actual risk of many genotoxic chemicals, natural constituents, contaminants and certain residues (e.g., pesticide metabolites and impurities) present in food and consumer products cannot be conclusively assessed due to a lack of substance-specific carcinogenicity data. In this respect, it is worth mentioning that the Threshold of Toxicological Concern (TTC) concept may be used to assess the toxicological relevance of (unintended) exposures to substances with a known chemical structure but insufficient toxicological data (EFSA 2012; EFSA 2019). However, in many cases, the relevant TTC for (suspected) DNA‐reactive mutagens of 0.0025 μg/kg body weight (bw) per day is exceeded, which means that a non‐TTC approach is still required to reach a conclusion (EFSA 2019).

In the absence of clear evidence for a threshold, the derivation of a health-based guidance value (HBGV), i.e., “a range of exposures (either acute or chronic) that are expected to be without appreciable health risk” (WHO/FAO 2020a), is generally not considered an option. Yet, with a better understanding of the various mechanisms leading to DNA damage and mutation, it became increasingly clear that certain modes of action (MoA) may justify a deviation from the default assumption of a non-threshold dose–response relationship (Bolt 2008; EFSA 2021; WHO/FAO 2020a). This applies in particular to purely aneugenic substances and other non-DNA-reactive genotoxicants, for which it is nowadays considered feasible to derive a HBGV. Moreover, in specific cases, with the required thorough mechanistic and quantitative understanding, HBGVs have also been proposed for well-studied DNA-reactive mutagens such as formaldehyde (Conolly et al. 2003, 2004).

## Discussed opportunities involving quantitative interpretation of genotoxicity data

In 2007, an exceptionally high contamination with the genotoxic substance ethyl methanesulfonate (EMS, CAS 62-50-0) was found in tablets of the HIV protease inhibitor Viracept (EMEA 2007a). In the further course of events, the European Medicines Agency’s Committee for Medicinal Products for Human Use (CHMP) called for non-clinical studies to be conducted to “better quantify the risk for patients who have been exposed to the contaminated drug” (EMEA 2007b). The responsible marketing authorization holder thereupon undertook considerable efforts to further characterize the toxicity of EMS, which included studies on the repeated-dose toxicity, the cross-species toxicokinetic behaviour and the in vivo single and repeated-dose genotoxicity in mice (Gocke et al. 2009; Lave et al. 2009; Pfister and Eichinger-Chapelon 2009). In a subsequent risk assessment, the authors concluded that there was sufficient evidence to support the assumption of a threshold dose–response relationship with respect to the clastogenic and mutagenic activities of EMS (Gocke and Müller 2009; Müller et al. 2009). This conclusion was also derived from earlier mechanistic observations suggesting that specific cellular DNA repair mechanisms are in place that can effectively remove O^6^-ethylguanine DNA adducts caused by EMS (Doak et al. 2007). Following this line of reasoning, Müller et al. (2009) identified an oral no-observed-effect level (NOEL) of 25 mg/kg bw/day based on *lacZ* mutant frequencies in the bone marrow and the gastrointestinal tract of the transgenic Muta^™^ Mouse. Finally, using this NOEL as a reference point, a MOE (“safety factor”) was calculated for the estimated maximum oral intake of exposed patients.

Notably, a large number of in vitro and in vivo studies, including mechanistic data—largely beyond the routine data requirements—were needed to support the assumption of a threshold for genotoxic effects of EMS. In addition, as pointed out by Lutz (2009), EMS is a small alkylating agent with low electrophilic reactivity. Thus, generalization of the data and postulation of thresholds for other genotoxic carcinogens without appropriate data and interpretation has been considered premature (Lutz 2009). Nonetheless, the experiences gained in connection with the Viracept contamination incident, as well as recent advances in the development of new testing methods, sparked a new discussion about the possibility of moving from qualitative hazard identification to a quantitative interpretation of genotoxicity data (Schuler et al. 2011; White et al. 2020). This new genotoxicity assessment paradigm was soon referred to as ‘quantitative genotoxicity’ in a growing number of scientific publications and its advancement became part of the agenda of several expert working groups (Gollapudi et al. 2013, 2011; Johnson et al. 2014; MacGregor et al. 2015a, 2015b; Schuler et al. 2011; White and Johnson 2016; White et al. 2020).

### Quantitative approaches for the analysis of genotoxicity data

Initial research on quantitative analysis of genotoxicity data focused on comparing different metrics for characterizing genotoxic potency, including the no-observed-genotoxic-effect level (NOGEL), the breakpoint dose (BPD) (also referred to as the threshold dose [Td]), and the BMD approach (Gollapudi et al. 2013; Johnson et al. 2014). In this context, the NOGEL has been defined as the highest tested dose for which no statistically significant increase in the genotoxic response was observed in relation to the untreated control (Gollapudi et al. 2013). Unlike the NOGEL, which always refers to an actual tested dose, the BPD is derived from the fitting of a bilinear model and refers to the dose that is associated with a break point in the fitted dose–response curve (Johnson et al. 2014; Lutz and Lutz 2009). The BMD approach, as already mentioned, is also based on the fitting of a mathematical model (or group of models) and represents an estimate of the dose that is associated with a given effect size (EFSA 2022; US EPA 2012). Among the different metrics examined, the BMD approach was consistently identified as the preferred method (Gollapudi et al. 2013; Johnson et al. 2014; MacGregor et al. 2015b). For example, the International Workshop on Genotoxicity Testing (IWGT) Working Group on Quantitative Approaches to Genetic Toxicology Risk Assessment (QWG) came to the following conclusion:

*The BMD approach was considered* *to be the preferred approach for dose(exposure)–response analysis and PoD derivation for genotoxicity data because: (1) dose–response analysis can be performed on studies with minimal data, (2) it uses the entire data set to derive a BMD estimate, (3) the size of the effect is defined, (4) covariate analysis can be performed, (5) within limits, the PoD value is not adversely affected by experimental design and dose selection (e.g., NOGELs from two different experiments can vary significantly due to differences in dose spacing and statistical sensitivity), (6) confidence limits can be derived. *(MacGregor et al. 2015b)*.*

After consensus was reached on the preferred method for analyzing dose–response relationships, questions relating to the biological implications of genotoxicity-based reference points increasingly came to the fore. For instance, the correlation between genotoxicity-based and carcinogenicity-based BMD estimates was studied to assess whether it is possible to predict carcinogenic potency from in vivo genotoxicity data (Hernandez et al. 2011; Soeteman-Hernandez et al. 2016). Due to the limited availability of suitable dose–response data, no general conclusion could be drawn from these meta-analyses of historical data. However, the comparison of dose–response data for a limited number of model compounds suggested a positive correlation between genotoxicity-based and carcinogenicity-based BMD estimates. Based on the relationships described above, an attempt was also made to predict the carcinogenicity of the furazolidone metabolite 3-amino-2-oxazolidinone using in vivo micronucleus data (RIVM 2014).

Another research focus has been the identification of appropriate critical effect sizes (CES) for the BMD modeling of genotoxicity data (Zeller et al. 2017, 2016). The CES, also referred to as the benchmark response (BMR), denotes a small but measurable change in a response that must be defined a priori in order to estimate a corresponding BMD. Initial BMD analyses of genotoxicity data often used fixed CES values of 5 or 10%. More recently, however, it has been noted that expert judgement may be required to select an appropriate CES for a given endpoint, and the use of higher CES values has been proposed in the context of modeling genotoxicity data (Jensen et al. 2019; Slob 2017; White et al. 2020; Zeller et al. 2016). In this sense, Zeller et al. (2017) derived endpoint-specific CES values from the (outlier-deprived) long-term distribution of historical vehicle control data. For most types of genotoxicity assays, this approach resulted in a CES that corresponded to an increase of ~ 50% over controls (Zeller et al. 2017). In another study, Chen et al. (2021) systematically compared critical effects sizes of 5, 10, 50 and 100% to quantify the adduct formation, the mutation frequency in kidney and the micronucleus formation in circulating reticulocytes in aristolochic acid exposed *gpt* delta mice. The higher CES values of 50 and 100% provided the most precise estimates measured as ratio between the upper and the lower limits of the 90% confidence interval. This might be linked to the better coverage of the effect range by the given data set—another consideration in CES selection. However, it should be noted that in several other BMD analyses of in vivo genotoxicity data, also lower CES values (5–10%) have resulted in precise BMD estimates (Cao et al. 2014; Duydu 2022; Gollapudi et al. 2013; Johnson et al. 2014).

There has also been vital interest in comparing BMD estimates between different compounds and genotoxicity endpoints, and also with respect to different covariates, such as sex, life stage, target tissue, cell type, assay variant, study duration, and tissue sampling time (Guerard et al. 2017; Long et al. 2018; Marchetti et al. 2021; Mittelstaedt et al. 2021; White et al. 2020; Wills et al. 2017, 2016a, 2016b). The results of these studies suggest that BMD modeling of both in vitro and in vivo genotoxicity data could serve as a useful metric for comparing the potency of substances in relation to a given assay. In addition, the BMD approach could be seen as a promising tool for sensitivity comparisons between different genotoxicity endpoints and for studying the influence of experimental covariates.

### Quantitative genotoxicity approaches in the regulatory context

While quantitative analyses of genotoxicity data are increasingly applied in non-regulatory contexts, their use in regulatory decision-making is still considered an exception from the rule (COM 2018). However, some expert working groups have expressed a general need for a paradigm shift (Gollapudi et al. 2013; Johnson et al. 2014; MacGregor et al. 2015a; White et al. 2020). In this context, the Quantitative Analysis Workgroup (QAW) of the Genetic Toxicology Technical Committee (GTTC) coordinated by the Health and Environmental Sciences Institute (HESI) found that “once genetic toxicology PoDs are calculated […], they can be used to derive reference doses and margin of exposure values that may be useful for evaluating human risk and regulatory decision making” (Johnson et al. 2014). Moreover, the IWGT QWG supported the quantitative interpretation of in vivo genotoxicity dose–response data “to determine PoDs to be used, with appropriate extrapolation methods and uncertainty factors, to establish regulatory exposure limits, and, in conjunction with human exposure data, to assess and manage the risk of adverse health effects” (MacGregor et al. 2015a).

#### Quantitative interpretation of genotoxicity data to calculate margin of exposure (MOE) values

Previous publications have discussed the MOE approach as an opportunity for quantitative interpretation of genotoxicity data in regulatory settings (Benford 2016; Johnson et al. 2014). However, different expert committees have expressed divergent opinions in this regard. As early as 2011, the EFSA Scientific Committee commented on the quantitative assessment of in vivo dose–response relationships: “In the future, such approaches may be compatible with the development of MOE approaches for genotoxicity data, especially if the collection of genotoxicity data is integrated into the standard toxicity tests” (EFSA 2011). The Committee on Mutagenicity of Chemicals in Food, Consumer Products and the Environment (COM), on the other hand, noted in 2018 that “the COM, at present, was unable to make any recommendations for the inclusion of quantitative genotoxicity data in MOE calculations” (COM 2018). Moreover, in view of recent developments and future directions, the authors of a recent publication by the World Health Organization (WHO) and the Food and Agriculture Organization (FAO) speculated that “lacking carcinogenicity data, quantitative analysis of in vivo mutagenicity dose–response data could be used for deriving MOEs” (WHO/FAO 2020a).

#### Quantitative interpretation of genotoxicity data to derive health-based guidance values (HBGVs)

Apart from MOE calculations, some authors suggested to consider genotoxicity data also for the derivation of HBGVs (Dearfield et al. 2017; Gollapudi et al. 2020; Heflich et al. 2020; Johnson et al. 2014; White et al. 2020). As already mentioned, a HBGV represents a range of exposures that are not expected to result in an appreciable health risk. For this reason, HBGVs are only derived for substances that cause effects for which a robust threshold can be determined (WHO/FAO 2020b). This applies, for example, to purely aneugenic substances for which the quantitative interpretation of genotoxicity data to identify a reference point and, on certain occasions, also to derive a HBGV is already considered an option (EFSA 2021). With regard to DNA-reactive substances, however, the consideration of genotoxicity data for estimating a threshold dose is controversial (WHO/FAO 2020a).

#### Quantitative interpretation of genotoxicity data to support MoA-based risk assessment of genotoxic carcinogens

Recently, MoA-based risk assessment concepts have been proposed to derive “science-based limit values” or “MoA-based thresholds” for certain types of genotoxic carcinogens (ECHA/RAC and SCOEL 2017; Hartwig et al. 2020). MoA-based risk assessment can be regarded as a flexible approach, which seeks to integrate detailed mechanistic knowledge and quantitative information on relevant key events (KEs) (e.g., induction of DNA damage and mutations) with respect to the substance (group) and apical outcome under investigation (Hartwig et al. 2020). Hence, the concept of MoA-based risk assessment is somewhat related to the concept of quantitative genotoxicity as both approaches involve the quantitative assessment of genotoxic effects. However, MoA-based risk assessment is in general only applicable to well-studied carcinogens for which the adverse outcome(s) (i.e., the carcinogenic effects), the underlying molecular mechanisms, and the quantitative relationships between key events of the investigated adverse outcome pathway (AOP) have been sufficiently characterized.

For example, an early MoA-based modelling of formaldehyde genotoxicity causing squamous cell carcinoma in vivo in the rat was presented by Conolly et al. (2003). This biologically inspired model took into account site-specific flux of formaldehyde from inhaled air into tissue using a three-dimensional fluid dynamics model of the rat nasal airways, assumed low dose linear dose–response for formation of DNA–protein cross-link (DPX) and either a J-shaped or a simple threshold dose–response for cytolethality causing regenerative cellular proliferation (CRCP) in the two-stage clonal growth model. Maximum likelihood methods were used to estimate parameter values based on experimental data for DPX formation and cell replication. As shown by likelihood calculations against rat cancer data, this exercise allowed to model in vivo risk in the dose range examined in the rat bioassays and may allow to provide MoA-based predictions of risk also for lower doses. The rat model was also translated to the human situation (Conolly et al. 2004). However, as shown later by Subramaniam et al. (2007) and Crump et al. (2008), the model(s) were extremely sensitive to parameter and model assumptions, highlighting the need for better input data and understanding of the biological effect network leading to the health outcome, both qualitatively and quantitatively in model organisms as well as the human population.

It is worth noting that several AOPs are currently under development that could provide a more advanced basis for quantitative modelling of genotoxic effects and downstream apical outcomes (Cho et al. 2022; SAAOP 2023). For example, AOP46 describes a mutagenic MoA leading to hepatocellular carcinoma (HCC), using Aflatoxin B1 (AFB1) as a case example, and AOP15 describes the alkylation of DNA in male pre-meiotic germ cells leading to heritable mutations. While established OECD test guideline studies can currently provide quantitative data as needed for establishing models of the entire AOP only for some of the relevant events (Table 1), research methods already exist for the majority of those events, at least in vitro.

**Table 1:** Examples of Adverse Outcome Pathways (AOPs) considered potentially useful for quantitative modelling of genotoxic key events and downstream apical outcomes Source: AOP-Wiki, SAAOP (2023)

AOP	Events
AOP15—Alkylation of DNA in male pre-meiotic germ cells leading to heritable mutations	MIE: Alkylation, DNA KE: inadequate DNA repair KE: increase, Mutations AO: increase, Heritable mutations in offspring
AOP46—AFB1: Mutagenic Mode-of-Action leading to Hepatocellular Carcinoma (HCC)	MIE: formation, Pro-mutagenic DNA Adducts KE: increased, Induced Mutations in Critical Genes KE: metabolism of Aflatoxin B1 (AFB1), Production of Reactive Electrophiles KE: clonal Expansion/Cell Proliferation, to form Altered Hepatic Foci (AHF) KE: increased, Insufficient repair or mis-repair of pro-mutagenic DNA adducts AO: tumorigenesis, Hepatocellular carcinoma
AOP296—Oxidative DNA damage leading to chromosomal aberrations and mutations	MIE: increase, Oxidative damage to DNA KE: inadequate DNA repair KE: increase, DNA strand breaks AO: increase, Mutations AO: increase, Chromosomal aberrations

Events: *MIE* molecular initiating events, *KE* key events, *AO* adverse outcomes

## Challenges arising from a quantitative interpretation of genotoxicity data

### Determination of reference points

By definition, a toxicological reference point “[…] is derived from experimental or observational data […]” and represents the “[…] lower bound on dose (upper bound on response) that corresponds to an estimated or predetermined low-effect level or no-effect level of the dose–response curve” (WHO/FAO 2020b). Thus, if a rather insensitive study is used to establish a reference point, the true potency of the substance will be underestimated. For this reason, to ensure that all relevant studies and toxicological endpoints are taken into account, the determination of a reference point usually involves a thorough evaluation of the entire hazard data (WHO/FAO 2020b). In addition, confidence in the overall assessment is often enhanced by the use of test methods that were designed to evaluate a wide range of effects throughout the entire organism, such as studies on subchronic toxicity (OECD TG 408), chronic toxicity (OECD TG 452/OECD TG 453), reproduction toxicity (OECD TG 416/OECD TG 443) or long-term carcinogenicity (OECD TG 451).

In contrast to the apical toxicity endpoints mentioned above, which are largely assessed by macroscopic and microscopic examination of entire organs, genetic toxicity is typically studied at the cellular or molecular level using specific markers for certain types of genetic damage. Measurements of genotoxicity markers at different sites in the body suggest that dose–response relationships can be tissue-specific. For example, oral exposure of the Muta^™^ Mouse model to polycyclic aromatic hydrocarbons (PAHs) induced more pronounced genotoxic effects in site-of-contact tissues than in hematopoietic tissues (Long et al. 2016). This was found to be likely a result of a combination of toxicokinetic aspects (i.e., absorption, distribution and metabolism) and tissue-specific factors such as DNA repair and cell proliferation (Long et al. 2016). In addition, as discussed in more detail below, current standard in vivo genotoxicity testing methods are limited in their ability to detect different types of genotoxic effects in multiple target tissues, cell populations and/or genomic locations. For these reasons, establishing a dose–response relationship for genotoxic effects that is representative of the organism as a whole is not trivial.

The in vivo micronucleus test according to OECD TG 474, for example, detects clastogenic and aneugenic events but is restricted to polychromatic erythrocytes sampled in the bone marrow and/or peripheral blood (OECD 2016a). In addition, though not yet fully validated, modified versions of the in vivo micronucleus test are available for the assessment of micronuclei induction in the liver and the gastrointestinal tract (EFSA 2021; Kirkland et al. 2019). The transgenic rodent (TGR) gene mutation assays according to OECD TG 488 have the advantage of being applicable to almost any target tissue. However, current TGR assays are restricted to the detection of selectable gene mutations in specific reporter transgenes (OECD 2020c). Moreover, the sampling time required to reach optimal sensitivity is tissue-specific and appears to be related to the turnover time of the studied cell population (Lambert et al. 2005). The mammalian erythrocyte *Pig-a* gene mutation assay, for which an OECD guideline (OECD TG 470) has recently been adopted (OECD 2022), is limited to the phenotypic detection of mutations in the endogenous phosphatidylinositol glycan, class A (*Pig-a*) gene. Up to now, the *Pig-a* gene mutation assay is only fully validated for erythrocytes sampled from peripheral blood, but it has also been adapted to germ cells and blood cells other than erythrocytes (Dobrovolsky et al. 2017; Ji and LeBaron 2017; Kirkland et al. 2019). The in vivo mammalian alkaline comet assay (OECD TG 489) can be applied to cells or nuclei isolated from many target tissues (OECD 2016b). However, the comet assay is limited to the detection of DNA strand breaks, and therefore, it is only considered as an indicator of mutagenic effects.

The characterization of uncertainties that could arise with the quantitative interpretation of genotoxicity data due to the technical limitations of current standard test methods is inherently challenging. Nevertheless, comparisons of BMD estimates obtained from different dose–response data sets for a given substance can at least provide a rough idea of the variability in sensitivity that occurs within the existing experimental boundaries. The available literature already provides several dose–response assessments of in vivo genotoxicity data that allow for comparisons of BMD estimates across different endpoints, target tissues and experimental settings (Cao et al. 2014; Chen et al. 2021; Gollapudi et al. 2013, 2020; Guerard et al. 2017; Johnson et al. 2014; Long et al. 2018; Marchetti et al. 2021; Mittelstaedt et al. 2021; White et al. 2020). As part of this review, we have compiled 104 BMD estimates for 10 substances from this body of literature (Supplementary Table 1). If a publication reported BMD estimates for multiple CES values, the CES which on average resulted in highest precision (i.e., the lowest BMDU/BMDL ratio) was selected. Moreover, 14 BMD estimates were excluded from the further analysis because the precision of the BMD estimate was rather low (i.e., BMDU/BMDL > 50) or the BMD was modeled from summary data without information on variability. Differences between BMD estimates are only statistically defensible if their confidence intervals (i.e., the BMDL-BMDU intervals) do not overlap (Wills et al. 2017). Therefore, a significant difference between dose–response relationships was only assumed when this condition was met. In addition, because it is usually not the point estimate of the BMD but the corresponding lower bound (BMDL) that is used as the reference point, we also examined differences between the lower bounds of significantly different BMD estimates. Pairwise comparisons between BMD estimates were made only for values obtained by a comparable modeling approach (i.e., identical CES and BMD source).

Exemplary comparisons of published BMD estimates (Tables 2, 3 and 4) confirm that results obtained from different dose–response data sets for a given substance can differ significantly. Furthermore, the available dose–response data suggest that differences between candidate reference points (i.e., BMDL values) across genotoxicity endpoints, even when related to the same target tissue, can easily span one order of magnitude or even more (Table 2). The same applies to BMDL values based on the same endpoint but determined from measurements in different target tissues (Table 3). Moreover, in some cases, the BMD estimate also appeared to be sensitive to the choice of experimental settings, such as exposure time or tissue sampling time (Table 4). Thus, based on the limited data available, it could be inferred that the outcome of a genotoxicity-based dose–response assessment depends to a considerable extent on the choice of endpoint(s), target tissue(s) and experimental settings. In this context, however, it is important to note that comparative BMD analyses performed by Wills et al. (2017) did not provide conclusive evidence that the choice of TGR assay variant had a significant impact.

**Table 2:** Exemplary pairwise comparisons of published in vivo genotoxicity benchmark dose (BMD) estimates across different endpoints

Substance	Data source	BMD source	Study type	Endpoint	Endpoint details	Tissue (cell type)	Experimental settings	CES (%)	BMDL	BMDU	BMDU/BMDL	BMDL ratio
1-Ethyl-1-nitrosourea	van Delft et al. (1998)	Johnson et al. (2014)	Mouse, single-dose, intraperitoneal	GM	*Dlb-1* MF	Small intestine	Exposure: day 1 Sampling: day 49	10	0.09	1.62	18.1	21.6
				GM	*lacZ* MF	Small intestine	Exposure: day 1 Sampling: day 49	10	1.94	10.09	5.2	
1-Ethyl-1-nitrosourea	Bhalli et al. (2011)	Johnson et al. (2014)	Mouse, single-dose, intraperitoneal	GM	*Pig-a* MF	Peripheral blood (RBCs)	Exposure: day 1 Sampling: day 28	10	0.12	0.63	5.3	11.3
				MN	–	Peripheral blood (RETs)	Exposure: day 1 Sampling: day 2	10	1.36	10.05	7.4	
1-Methyl-1-nitrosourea	Monroe et al. (1998)	Johnson et al. (2014)	Mouse, single-dose, intraperitoneal	GM	*hprt* MF	Spleen	Exposure: day 1 Sampling: day 21	10	2.06	4.78	2.3	2.6
				GM	*LacI* MF	Spleen	Exposure: day 1 Sampling: day 21	10	5.26	16.25	3.1	
Aristolochic acid I	Chen et al. (2021)	Chen et al. (2021)	Mouse, repeated-dose, oral	GM	*gpt* MF	Kidney	Exposure: days 1–28 Sampling: day 31	50	0.40	0.68	1.7	6.0
				MN	–	Peripheral blood (RETs)	Exposure: days 1–28 Sampling: day 29	50	2.41	6.25	2.6	
Benzo[a]pyrene	Long et al. (2018)	Long et al. (2018)	Mouse, repeated-dose, oral	GM	*lacZ* MF	Small intestine	Exposure: days 1–28 Sampling: day 31	100	0.74	1.03	1.4	10.5
				MN	–	Peripheral blood (RETs)	Exposure: days 1–28 Sampling: day 30	100	7.75	13.1	1.7	
Ethyl methanesulfonate	Cao et al. (2014)	Cao et al. (2014)	Mouse, repeated-dose, oral	GM	*gpt* MF	Spleen	Exposure: days 1–28 Sampling: day 31	10	0.35	2.0	5.8	19.4
				MN	–	Peripheral blood (RETs)	Exposure: days 1–28 Sampling: day 13	10	6.79	8.84	1.3	
Temozolomide	Guerard et al. (2017)	Guerard et al. (2017)	Rat, repeated-dose, oral	MN	–	Peripheral blood (RETs)	Exposure: days 1–5, 57–59 Sampling: day 59	100	0.3	1.8	6.0	9.7
				DNA SB	comet TI	Liver	Exposure: days 1–5, 57–59 Sampling: day 59	100	2.9	6.8	2.3	

BMD estimates are reported as the lower bound (BMDL) and the upper bound (BMDU) of the BMD in mg/kg bw/day (repeated-dose oral exposure) and mg/kg bw (single-dose intraperitoneal exposure). Critical effect size as selected by the study authors of the referenced BMD source

*GM* gene mutation, *MN* micronucleus formation, DNA SB, DNA strand breaks, *MF* mutant frequency, *TI* tail intensity, *RBCs* mature red blood cells, *RETs* reticulocytes, *CES* critical effect size

**Table 3:** Exemplary pairwise comparisons of published in vivo genotoxicity benchmark dose (BMD) estimates across different target tissues

Substance	Data source	BMD source	Study type	Endpoint	Endpoint details	Tissue (cell type)	Experimental settings	CES (%)	BMDL	BMDU	BMDU/BMDL	BMDL ratio
Benzo[a]pyrene	Long et al. (2018)	Long et al. (2018)	Mouse, repeated-dose, oral	GM	*lacZ* MF	Small intestine	Exposure: days 1–28 Sampling: day 31	100	0.74	1.03	1.4	11.5
						Liver	Exposure: days 1–28 Sampling: day 31	100	8.47	14.6	1.7	
Ethyl methanesulfonate	Cao et al. (2014)	Cao et al. (2014)	Mouse, repeated-dose, oral	GM	*gpt* MF	Spleen	Exposure: days 1–28 Sampling: day 31	10	0.35	2.0	5.8	6.6
						Liver	Exposure: days 1–28 Sampling: day 31	10	2.3	14.95	6.5	
Ethyl methanesulfonate	Gocke and Wall (2009)	Gollapudi et al. (2013)	Mouse, repeated-dose, oral	GM	*lacZ* MF	Bone marrow	Exposure: days 1–28 Sampling: day 31	10	9.29	36.84	4.0	4.4
						Liver	Exposure: days 1–28 Sampling: day 31	10	41.0	98.12	2.4	

BMD estimates are reported as the lower bound (BMDL) and the upper bound (BMDU) of the BMD in mg/kg bw/day. Critical effect size as selected by the study authors of the referenced BMD source

*GM* gene mutation, *MF* mutant frequency, *CES* critical effect size

**Table 4:** Exemplary pairwise comparisons of published in vivo genotoxicity benchmark dose (BMD) estimates across different experimental settings

Substance	Data source	BMD source	Study type	Endpoint	Endpoint details	Tissue (cell type)	Experimental settings	CES (%)	BMDL	BMDU	BMDU/BMDL	BMDL ratio
1-Methyl-1-nitrosourea	Bristol–Myers Squibb, unpublished data	Johnson et al. (2014)	Rat, repeated-dose, oral	GM	*Pig-a* MF	Peripheral blood (RBCs)	Exposure: days 1–28 Sampling: day 29	10	0.015	0.13	8.7	13.3
							Exposure: days 1–28 Sampling: day 15	10	0.2	0.3	1.5	
1-Methyl-1-nitrosourea	Lynch et al. (2011)	Johnson et al. (2014)	Rat, repeated-dose, oral	MN	–	Peripheral blood (NCEs)	Exposure: days 1–28 Sampling: day 29	10	0.13	0.29	2.2	5.6
							Exposure: days 1–28 Sampling: day 4	10	0.73	2.28	3.1	
Aristolochic acids	Bhalli et al. (2013)	White et al. (2020)	Rat, repeated-dose, oral	GM	*Pig-a* MF	Peripheral blood (RBCs)	Exposure: days 1–28 Sampling: day 56	100	0.47	0.87	1.9	12.4
							Exposure: days 1–3 Sampling: day 29	100	5.83	9.64	1.7	
Ethyl methanesulfonate	Cao et al. (2014); Mittelstaedt et al. (2021)	Mittelstaedt et al. (2021)	Mouse, repeated-dose, oral	GM	*gpt* MF	Kidney	Life stage: neonate exposure: days 1–28 Sampling: day 31	50	6.62	16.65	2.5	2.8
							Life stage: adult exposure: days 1–28 Sampling: day 31	50	18.74	40.05	2.1	
Ethylene oxide	Donner et al. (2010)	Gollapudi et al. (2020)	Mouse, repeated-dose, inhalation	CA	RT	Peripheral blood (LYMs)	Exposure: weeks 1- 48 Sampling: week 49	50	1.84	11.8	6.4	11.1
							Exposure: weeks 1–12 Sampling: week 13	50	20.5	44.8	2.2	
Methyl methanesulfonate	Dertinger et al. (2012)	White et al. (2020)	Rat, repeated-dose, oral	GM	*Pig-a* MF	Peripheral blood (RBCs)	Exposure: days 1–28 Sampling: day 42	100	2.9	6.68	2.3	5.3
							exposure: days 1–3 Sampling: day 15	100	15.4	34.3	2.2	
Temozolomide	Guerard et al. (2017)	Guerard et al. (2017)	Rat, repeated-dose, oral	GM	*Pig-a* MF	Peripheral blood (RBCs)	Exposure: days 1–5, 57–59 Sampling: day 44	100	0.13	3.6	27.7	60.8
							Exposure: days 1–5, 57–59 Sampling: day 29	100	7.9	60.4	7.6	

BMD estimates are reported as the lower bound (BMDL) and the upper bound (BMDU) of the BMD in mg/kg bw/day (repeated-dose oral exposure) and parts per million (inhalation exposure). Critical effect size as selected by the study authors of the referenced BMD source

*GM* gene mutation, *MN* micronucleus formation, *CA* chromosomal aberrations, *MF* mutant frequency, *RT* reciprocal translocation, *RBCs* mature red blood cells, *NCEs* normochromatic erythrocytes, *LYMs* lymphocytes, *CES* critical effect size

It should further be noted that BMD modeling in general and also specifically for genotoxicity data has evolved over the years, and the older BMD analyses discussed here may not be fully in line with current recommendations. However, we believe that these data are still adequate for a rough comparison of dose–response relationships. The precision of BMD estimates, and possibly the convergence of BMDL values determined from different data sets for the same substance, could potentially be further improved by optimizing genotoxicity studies for dose–response analysis (e.g., by increasing the number of dose groups). It should be kept in mind, however, that the current in vivo genotoxicity test methods are still limited to specific types of effects and/or target tissues. Therefore, the overall difference in sensitivity between tissues of exposed animals may be even larger than indicated by the reviewed dose–response data.

Note that, in principle, human epidemiological data may also be used to establish a toxicological reference point (WHO/FAO 2020b). However, the quantitative interpretation of genotoxicity markers currently available for human samples (e.g., micronuclei and DNA strand breaks in peripheral blood) would be subject to essentially the same limitations as described above for animal experimental data.

### Calculation of margin of exposure (MOE) values

The calculation of MOE values from genotoxicity-based reference points has been discussed in previous publications as an opportunity to support regulatory decision-making (Benford 2016; Johnson et al. 2014; WHO/FAO 2020a). However, a possible adoption of this approach into regulatory practice may first require a thorough evaluation of the extent to which genotoxicity-derived MOE values can provide a reliable characterization of health risks. Since MOE calculations for genotoxic substances are traditionally based on reference points from a carcinogenicity study, comparing genotoxicity-based and carcinogenicity-based BMD estimates seems to be a pragmatic and straightforward approach to assess whether consideration of genotoxicity as a surrogate endpoint would considerably alter the assessment outcome.

In a meta-analysis of published dose–response data for 18 different compounds, Hernandez et al. (2011) compared genotoxicity-based BMD_10_ estimates from the in vivo micronucleus assay and the TGR assay with BMD_10_ estimates from carcinogenicity studies in mice. Interestingly, although BMDs were compared across different routes of exposure, a positive correlation was observed between tissue-matched BMD_10_ values when the lowest BMD_10_ value from both micronucleus and TGR data was taken into account. However, the correlation was considerably lower when examined either for micronucleus or TGR data alone. More importantly, a poor correlation was observed when the lowest genotoxicity-based BMD_10_ value was plotted against the lowest (i.e., non-tissue-matched) carcinogenicity-based BMD_10_ value. In a second study, the association between genotoxicity-based and carcinogenicity-based BMD values was further investigated for a larger data set consisting of 48 model compounds (Soeteman-Hernandez et al. 2016). In this case, obtained scatter plots indicated a moderate correlation between the lowest BMD_05_ values from in vivo micronucleus data and the lowest BMD_10_ values for carcinogenic effects, but there was still a considerable scatter in the data spanning roughly a factor of 100 in both directions. It is worth noting that rather low CES values were used to obtain BMD estimates from genotoxicity data. However, according to the authors, the choice of the CES had no crucial influence on the results in either of the two meta-analyses (Hernandez et al. 2011; Soeteman-Hernandez et al. 2016). The main purpose of the first meta-analysis was to investigate whether a correlation can be established at all (Hernandez et al. 2011). In the second meta-analysis, constant shape parameters were used and therefore all BMD estimates would have been increased by a constant factor if a higher CES had been selected (Soeteman-Hernandez et al. 2016).

In a recent study by Chepelev et al. (2023), the above comparisons of in vivo genotoxicity and carcinogenicity dose–response data were expanded through the additional consideration of human exposure data to enable a direct comparison of the resulting MOE values and their theoretical regulatory implications. The use of a genotoxicity-based reference point (micronucleus induction, BMDL_05_) in connection with upper bound exposure estimates resulted in MOE values below 10,000 for 15 of the 48 evaluated model compounds, whereas the corresponding carcinogenicity-based reference points (BMDL_10_) yielded MOE values below 10,000 in 13 of 48 cases. The genotoxicity-derived MOE values were still below 10,000 in 11 out of 48 cases when the CES for the analysis of the micronucleus data was increased to 50%. Based on these findings, the authors concluded that regulatory decisions based on in vivo genotoxicity dose–response data would be consistent with those based on carcinogenicity dose–response data. However, these findings should be interpreted with caution since the above distributions of categorical assessment outcomes (i.e., MOE > 10,000 or MOE < 10,000) are likely to be highly sensitive to changes in the exposure data. A direct comparison of genotoxicity-derived (micronucleus induction, BMDL_05_) and carcinogenicity-derived MOE values rather shows that these would not always be consistent: In 20 out of 48 cases corresponding MOE values differed by more than one order of magnitude and in 8 out of 48 cases even by two orders of magnitude. In 41 out of 48 cases, the genotoxicity-derived MOE (micronucleus induction, BMDL_05_) was lower than the carcinogenicity-derived MOE.

The above described comparisons of BMD estimates and subsequently derived MOE values suggest that the use of genotoxicity data would in most cases lead to a more conservative assessment result (assuming that the same MOE is used to identify a low level of concern). However, in some cases, the genotoxicity-based reference point (and thus also the calculated MOE value) may differ by up to two orders of magnitude from the corresponding carcinogenicity-based value. So far, the exact reasons for these large deviations are not fully clear. On the one hand, genotoxic potency, at least as currently determined, may simply not be a very accurate predictor of carcinogenic potency. On the other hand, (ignored) differences in other variables such as species, strain, exposure time, and route of administration may have resulted in a high proportion of unexplained variability. In addition, since the comparative analyses were largely based on in vivo micronucleus data, it is likely that substance-specific differences in systemic bioavailability and thus target tissue exposure had a significant impact on the studied correlations. Therefore, it could be speculated that a better correlation could probably be established between genotoxicity-based and carcinogenicity-based reference points (and also between the derived MOE values) if more comprehensive and standardized genotoxicity dose–response data were available for analysis.

Regarding the regulatory interpretation of genotoxicity-derived MOE values, additional challenges may arise from the fact that DNA damage and mutation are early events in the multistep process of carcinogenesis and that only a minor fraction of these early events is expected to result in uncontrolled cell proliferation and ultimately the development of a tumor. Based on this reasoning, it has been speculated that measurements of early genotoxic events in the pathway to carcinogenesis would generally result in reference points lower than those identified from tumor incidences (MacGregor et al. 2015a). Due to a lack of standardized data, it has not yet been possible to test this assumption for a representative number of substances. However, in case of 2-amino-3,8-dimethylimidazo[4,5-f]quinoxaline (MeIQx), which was tested for mutagenicity and carcinogenicity in rats under comparable study conditions, the BMD_10_ for *lacI* mutant frequency in the liver was indeed lower than the BMD_10_ for liver hepatocellular adenoma and carcinoma (MacGregor et al. 2015a). This would also be consistent with the general trend towards more conservative assessment results seen in the comparisons of genotoxicity- and carcinogenicity-based MOE values (Chepelev et al. 2023). Consequently, even if a good correlation between genotoxicity-based and carcinogenicity-based reference points could be established, MOE values calculated from genotoxicity endpoints may still require a different regulatory interpretation. Additional data may be needed to evaluate whether it is possible to define a magnitude of the MOE that can be considered indicative of a low level of concern.

### Derivation of health based guidance values (HBGVs)

Apart from the calculation of MOE values, the derivation of HBGVs has been discussed as another option to consider genotoxicity-based reference points in regulatory decision-making (Dearfield et al. 2017; Heflich et al. 2020; Johnson et al. 2014; White et al. 2020). As compared to the MOE, which is often used to identify and communicate health concerns in the context of unintended or unavoidable exposure to genotoxic carcinogens, a HBGV has much more far-reaching regulatory implications. Fewer restrictions usually apply to substances for which a HBGV can be derived. For this reason, a HBGV, i.e., “a range of exposures (either acute or chronic) that are expected to be without appreciable health risk” (WHO/FAO 2020b), should only be derived if this is supported with reasonable certainty by the available data.

As the default assumptions on thresholds are different for non-DNA reactive and DNA reactive agents, a meaningful discussion on the suitability of genotoxicity data to derive a HBGV is only possible if the substance-specific MoA is taken into account. Regarding genotoxic substances with a non-DNA-reactive MoA, it is generally accepted that an identifiable threshold may exist (Bolt 2008; EFSA 2021; WHO/FAO 2020a). In such cases, derivation of an HBGV by well-established principles, e.g., according to WHO/FAO (2020b), is generally considered an option. This means that dose–response relationships for all relevant toxicity endpoints would need to be evaluated prior to the derivation of an HBGV. Hence, for non-DNA-reactive substances, the additional consideration of in vivo genotoxicity data would provide an extension of the data basis that might even increase confidence in the derived HBGV. However, it remains open how to proceed if the reference point for genotoxicity is substantially lower than the conventionally derived reference point. For aneugenic substances, EFSA recommends to establish an acute HBGV based on aneugenicity data by applying the common 100-fold default uncertainty factor (UF) (i.e.*,* 10 for interspecies and 10 for intraspecies differences) in this case. Moreover, an additional UF may be applied using expert judgment, for example to account for sensitivity differences between somatic cells and germ cells (EFSA 2021).

For substances with a DNA-reactive MoA, the discussion is inherently more complex. First, the question arises whether the default assumption of a non-threshold dose–response relationship is at all compatible with the derivation of an HBGV. In view of the internationally recognized definition of the term HBGV, any such procedure would inherently require a preceding discussion on what increase of risk should be regarded as “not appreciable” and how this increase in risk could be related to a measurable (genotoxicity) endpoint (Neumann 2009). Eventually, this leads us to the not-so-new problem of defining an increase of risk that is societally deemed acceptable. However, this is foremost a policy and societal question, which should further involve questions regarding the objective to be protected from which harm (e.g. risk–benefit in pharmaceutical sector or protection of human health following intentionally added active substances in pesticide legislation).

For certain types of DNA-reactive carcinogens, the possibility of a MoA-based risk assessment to establish “science-based limit values” has been discussed (Hartwig et al. 2020). One of the most prominent examples in this regard is the alkylating substance EMS. There is a large body of experimental data from cell lines and animal models suggesting that O^6^-alkylguanine DNA adducts, including O^6^-ethylguanine adducts induced by EMS, are effectively removed through the DNA repair enzyme O^6^-methylguanine methyltransferase (MGMT). However, since MGMT removes O^6^-alkylguanine adducts in a suicide reaction, the enzyme becomes depleted at higher doses (Thomas et al. 2015). Based on this mechanistic information, together with evidence from in vitro as well as in vivo genotoxicity studies supporting a non-linear dose–response relationship, it was assumed that the induction of mutations by EMS follows a threshold-like dose–response relationship and a NOEL for the oral route was proposed (Doak et al. 2007; Gocke and Müller 2009; Müller et al. 2009).

It should be noted that even in situations where a mechanistic threshold may appear plausible under certain experimental conditions, establishing a HBGV for the general population would still be associated with considerable uncertainties (WHO/FAO 2020a). As discussed in Section “Hazard characterization”, studies focusing on downstream apical endpoints such as reproductive toxicity and carcinogenicity are very limited in their ability to detect adverse events in the human relevant dose range. Thus, for DNA-reactive substances, the derived HBGV would likely be highly dependent on the genotoxicity test results. This is somewhat problematic because, as outlined in Section “Determination of reference points”, in vivo genotoxicity dose–response relationships can strongly depend on the choice of endpoint(s), target tissue(s) and experimental settings. In addition, unlike studies on reproductive toxicity and carcinogenicity, in vivo genotoxicity is often assessed only in one rodent species (mouse or rat) and over a relatively short period of time. The OECD test guidelines for reproductive toxicity and carcinogenicity, on the other hand, recommend the use of rabbits and rats, or mice and rats, respectively, and also provide for longer treatment durations and the inclusion of particularly sensitive developmental stages. These differences in the principles and initial conception of the relevant OECD test guidelines further complicate and hamper the use of genotoxicity data for HBGV derivation.

It also needs to be considered that protective metabolic and DNA repair pathways are generally subject to high variability, as their capacity is influenced by various factors, including genetics, age, nutrition, lifestyle, diseases, and the environment (EFSA 2005; Hartwig et al. 2002; Karakaidos et al. 2020; Niazi et al. 2021; Pachkowski et al. 2011; Tyson and Mathers 2007). For example, there is evidence that the acquisition of deficiencies in DNA repair (including MGMT) in large areas of a tissue, e.g., as a result of epigenetic downregulation of DNA repair genes (“field defects”), is likely to play a central role in the development of some human cancers (Bernstein et al. 2013; Katsurano et al. 2012; Svrcek et al. 2010). Indeed, as discussed in White et al. (2020), deficiencies in DNA repair or lesion bypass mechanisms can substantially increase the susceptibility of exposed cells to genotoxicants. For example, MGMT-deficient cells exposed to alkylating agents in vitro showed a 4–16-fold increase in susceptibility compared to the corresponding wild-type cells depending on the studied endpoint and exposure conditions.

Additional challenges arise from the fact that multiple genotoxic substances can collectively affect the integrity of the genome and that dose–response relationships of many genotoxic agents are modulated by the same metabolic enzymes and/or DNA repair pathways. Consequently, even if an exposure level could be identified for a single substance at which protective mechanisms would not be overwhelmed, a reduction in the capacity to respond to other genotoxic stressors would still be a consequence. For instance, combined genotoxic effects have been demonstrated in vitro for the alkylating agents methyl methanesulfonate and ethyl methanesulfonate (Kojima et al. 1992) and also for a polycyclic aromatic hydrocarbon (benzo*[a]*pyrene) and a heterocyclic amine (2-amino-1-methyl-6-phenylimidazo[4,5-b]pyridine) (Jamin et al. 2013). Moreover, in vitro and in vivo mutagenicity studies on mixtures of polycyclic aromatic hydrocarbons supported the assumption of dose additivity (Lemieux et al. 2015; Long et al. 2017).

Finally, it should be noted that the presence of uncertainties per se is not an argument against the derivation of a HBGV. Even the traditional approach is not free of uncertainties and the use of UFs to account for possible differences in sensitivity has a long tradition in regulatory toxicology. Revisiting the concept of uncertainty factors, White et al. (2020) proposed data-derived UFs to be considered in addition to the default UF of 100 in order to account for possible deficiencies in compensatory pathways (UF 2–20) and also for short (i.e., less than 28 days) treatment durations (UF 5–15). However, additional data may be needed to better characterize the numerous sources of uncertainty that would be associated with the derivation of HBGVs from genotoxicity data.

## Perspectives

Where does this discussion leave us? Over several decades, the presence or absence of molecular effect thresholds for genotoxicity but also for the more complex endpoint of carcinogenicity has been subject to lively debate (Neumann 2009). In this context, the integration of detailed information on the genotoxic and/or carcinogenic MoA is increasingly regarded as an opportunity to refine regulatory assessment and classification schemes (Bolt 2008; Doe et al. 2022; ECHA/RAC and SCOEL 2017; Felter et al. 2021; Hartwig et al. 2020). However, within the rather practical remit of regulatory toxicology and public health protection, the question is not only if there is a mechanistically plausible threshold for a particular genotoxic substance but also whether this threshold can be determined precisely enough to allow reliable conclusions to be drawn about the presence or absence of health concerns. As outlined above, this has traditionally not been the case, particularly for DNA-reactive genotoxicants but also for many of the non-DNA-reactive ones.

While the current “binary” approach has its regulatory benefits and also fits a bill of precaution, it is certainly not free of downsides. In many situations, the exposure to genotoxic substances cannot completely be avoided through regulatory measures. For example, we knowingly come into contact with genotoxicants as well as carcinogens on a daily basis when consuming certain foods such as (fried) potatoes, coffee, alcoholic beverages, soy products, various vegetables or some spices. These scenarios of unintended or unavoidable exposure often cannot be assessed conclusively by means of traditional assessment schemes. While this can and should be no reason for completely questioning the current regulatory approach, it seems obvious that there is still room for improvement. This concerns in particular the characterization of health effects at low, human relevant exposures.

### Lessons to be learned from case studies

Case studies focusing on the quantitative risk assessment of a specific genotoxic substance are certainly useful to identify practical hurdles and research needs. For instance, recent case studies on benzene (Luijten et al. 2020), ethylene oxide (Gollapudi et al. 2020) and N-nitrosamines (Johnson et al. 2021) explored the use of in vivo genotoxicity dose–response data in connection with preliminary UFs to derive levels of “acceptable daily exposure” or “permitted daily exposure”. An important finding of these case studies was that there is a lack of consensus on which UFs should be used to derive exposure limits from genotoxicity-based reference points. However, apart from the issue that a simple threshold approach may not be scientifically defensible for most DNA-reactive substances (as already discussed in Section “Derivation of health based guidance values (HBGV)”), it should be noted that establishing science-based UFs for the interpretation of genotoxicity-based reference points is generally difficult. This is mainly due to the fact that the magnitude of uncertainty to be covered is still poorly characterized. As discussed in Section "Determination of reference points" , a major portion of the uncertainty arising from the quantitative interpretation of genotoxicity data is probably attributed to the limitations of current in vivo genotoxicity test methods. Therefore, rather than just further exploring the quantitative interpretation of existing genotoxicity data, priority should be given to the development of advanced experimental tools that allow for a more comprehensive evaluation of dose–response relationships, as briefly discussed in the following chapters.

### Integration of genotoxicity endpoints into standard repeated-dose toxicity studies is considered to be an important step in the advancement of in vivo genotoxicity testing and quantitative assessment of dose–response data

For several reasons, the integration of genotoxicity endpoints into standard repeated-dose toxicity studies is considered an important step in the advancement of in vivo genotoxicity testing and quantitative assessment of dose–response data. The combined evaluation of genotoxicity and repeated-dose toxicity not only aids the interpretation of genotoxicity findings (e.g., identification of relevant target tissues) by generating concurrent information on toxicokinetics, haematology, clinical chemistry and histopathology (Dertinger et al. 2010; EFSA 2011; Rothfuss et al. 2011). It can also help to reduce the need for test material, animals and other resources (Hamada et al. 2001; Krishna et al. 1998) which can also improve the general availability of genetic toxicology dose–response data. For a variety of genotoxicity test methods, including the in vivo micronucleus test, the comet assay and the *Pig-a* gene mutation assay, an integration into standard repeated-dose toxicity studies is nowadays considered technically feasible (Dertinger et al. 2010; Hamada et al. 2001; Shi et al. 2011). The integration of gene mutation studies for tissues other than blood is somewhat more complicated because, unlike standard repeated-dose toxicity studies, these are usually performed in transgenic rodent models. Provided that a transgenic model with appropriate genetic background is used, TGR assays could theoretically also be combined with standard repeated-dose toxicity and carcinogenicity studies (Akagi et al. 2015; Dobrovolsky and Heflich 2018). However, since current TGR assays are generally labor intensive and expensive, their integration into repeated-dose toxicity studies on a routine basis is rather unlikely (Dobrovolsky and Heflich 2018). Consequently, there is certainly a need to develop more efficient and less constrained methods for the in vivo detection of gene mutations that can also be readily integrated into standard repeated-dose toxicity studies.

### Error-corrected NGS (ecNGS) techniques could help to overcome some of the technical hurdles currently preventing a stronger consideration of genetic toxicity dose–response data

Error-corrected next-generation sequencing (ecNGS) is increasingly recognized as a promising technique to study the mutagenic effects of chemicals in vivo (Du et al. 2017; Heflich et al. 2020; LeBlanc et al. 2022; Marchetti et al. 2023; Maslov et al. 2015; Salk and Kennedy 2020; Valentine et al. 2020). Due to the relatively high error rate generated during library construction and sequencing, conventional next-generation sequencing (NGS) technologies can only be used for studying mutations in the germline or in tumor clones (Du et al. 2017). In contrast, recently developed ecNGS techniques, such as single cell sequencing, duplex sequencing, or circle sequencing, enable the detection of rare somatic mutations in primary cells and tissues at a frequency even lower than 1 × 10^–7^ mutations per nucleotide site (Du et al. 2017; Maslov et al. 2015). Moreover, unlike the currently used TGR assays, ecNGS techniques are not constrained to transgenic animal models (Salk and Kennedy 2020). This opens up completely new possibilities for a comprehensive assessment of mutagenic effects within repeated-dose toxicity studies. Valentine et al. (2020) successfully applied a duplex sequencing approach to detect low-abundant mutations in five tissues of two strains of mice after repeated exposure to three selected carcinogens (benzo[a]pyrene, N-ethyl-N-nitrosourea and urethane). In Big Blue C57BL/6 mice treated either with benzo[a]pyrene or N-ethyl-N-nitrosourea, a good overall correlation of fold increases in mutant frequency at the *cII* locus determined independently by duplex sequencing and the conventional TGR assay was observed. Based on these findings, the authors concluded that ecNGS techniques “can be used to rapidly detect and quantify the in vivo mutagenic impact of environmental exposures or endogenous processes in any tissue, from any species, at any genomic location” (Valentine et al. 2020). Additional advantages result from the fact that ecNGS-based approaches provide a direct and detailed characterization of the genetic material down to the base-pair level. Hence, the method is not restricted to mutations that produce a selectable phenotype and valuable additional information on mutation spectra and mutation signatures can be extracted from the sequence data with minimal effort (Du et al. 2017; Maslov et al. 2015; Valentine et al. 2020). In conclusion, ecNGS methods could indeed help to overcome some of the technical hurdles currently preventing a stronger consideration of genetic toxicity dose–response data.

### Integration of quantitative information on key events (KE) along well-established adverse outcome pathways (AOPs) should allow more informed assessment of the likely effect sizes at low, human relevant exposures

The current suite of standardized OECD test guideline methods for genotoxicity assessment provides only very limited information on the substance-specific MoA. Thus, complementing existing standard test methods with assays targeting at the underlying mechanisms is an important step towards the development of MoA-based quantitative models. Moreover, as discussed above, chemical agnostic AOPs for genotoxicity have already been proposed and are further developed to provide the required framework for such studies (SAAOP 2023). Assays addressing the KEs of those AOPs either directly or indirectly through suitable biomarkers will allow to assign chemicals under investigation to one or more AOP, also assisting in the use of knowledge about other chemicals triggering the same AOP to inform its risk assessment.

Discussions are already ongoing at OECD level regarding the possible development of respective test guidelines: One technology of interest is the proprietary ToxTracker^®^ making use of reporter gene assays addressing biomarkers for oxidative stress (Srxn1, Blvrb), DNA damage (Bscl2, Rtkn), cellular and protein damage (Btg2, Ddit3) (Smart et al. 2022). The γH2AX assay is discussed at OECD level, measuring phosphorylation of histone H2AX which represents a biomarker of DNA double strand breaks (DSBs) and may provide a quantitative description of the dose–response (OECD 2018). Combining the γH2AX assay with another histone biomarker (pH3) was also shown to be useful to efficiently discriminate between aneugenic and clastogenic compounds (Kopp et al. 2019). With respect to the potential contribution of such methods to the development of MoA-based risk assessment models, it may be worth noting that the induction of DNA DSBs has been identified as an intermediate key event in a proposed AOP for the development of infant leukemia, linking interference with topoisomerase II to rearrangements of the mixed-lineage leukemia (*MLL*) gene (Pelkonen et al. 2017). Methods for measuring topoisomerase inhibition in chemico, in vitro and in vivo have also been made available, for example, by Nitiss et al. (2021).

A concept has been developed to link the size of the effect at such a molecular initiating event (MIE) or early key events to those at the downstream key events within an AOP including the apical adverse outcome through quantitative key event relationships (qKERs), ultimately building quantitative AOPs (qAOPs) (Conolly et al. 2017; Paini et al. 2022; Perkins et al. 2019; Spinu et al. 2020). There is great promise that the knowledge that may become accumulated in those qAOPs over the coming years might allow us to extrapolate from the interaction between a genotoxic substance and its target or changes at a key event, which can be measured precisely over a broad range, to the size of the apical effect. At least, this knowledge should underpin assumptions about the existence of a threshold for genotoxicants assigned to a certain (q)AOP or the shape of the overall dose–response curve in different tissue and species, which is key to setting appropriate margins of exposure. In other words, integration of the quantitative dose–response information along an AOP should allow more informed assessment of the likely effect sizes at low, human relevant exposures.

The development of AOPs linking genotoxic key events to adverse health outcomes may also create new opportunities for the use of new approach methodologies (NAMs) and the further development of integrated approaches to genotoxicity testing and assessment. In this context, recent advances in the development of higher throughput in vitro genotoxicity assays can provide an important impetus. For example, a multi-endpoint, higher-throughput, in vitro genotoxicity assessment platform, termed GeneTox21, has recently been established (Fortin et al. 2023; Hanna et al. 2020; Long et al. 2019). The GeneTox21 platform consists of the Ames fluctuation (Ames II) and MutaMouse FE1 cell mutagenicity assays, the MicroFlow^®^ micronucleus assay, the MultiFlow^®^ DNA damage response biomarker assay, the CometChip^®^ DNA strand-break assay, and the TGx-DDI gene expression assay. This combination of assays is expected to reliably identify genotoxic substances and provide valuable MoA information. In addition, concentration–response data obtained from these higher-throughput genotoxicity NAMs were submitted to a high-throughput in vitro-to-in vivo extrapolation (IVIVE) model to derive an administered equivalent dose (AED), which in turn served as the starting point for the calculation of a bioactivity-exposure ratio (BER) (Beal et al. 2023; Kuo et al. 2022). Although any conclusions regarding the biological relevance and regulatory applicability of such AED values would be premature at this stage, this NAM-based approach could well describe a possible way forward towards a more efficient identification, characterization, and possibly prioritization of genotoxic chemicals.

## Conclusions

The quantitative interpretation of genotoxicity data to support regulatory decision-making is currently limited to few exceptions (e.g., purely aneugenic substances). Yet, whether and how genotoxicity should be regarded as an appropriate endpoint for the determination of reference points to be used in quantitative risk assessment also for other classes of genotoxicants (e.g., DNA-reactive substances) is still controversial. In this context, currently discussed opportunities mainly include the determination of a reference point (e.g., a benchmark dose), followed by calculation of a MOE or derivation of a HBGV.

In addition to new opportunities, major challenges arise from the quantitative interpretation of genotoxicity data. These challenges are mainly attributed to:

- The need to extrapolate a dose–response relationship that is representative for the organism as a whole from site-specific measurements of genotoxicity markers at the cellular or molecular level,
- The limitations of currently available standard methods for in vivo genotoxicity testing with regard to the detection of different types of genotoxic effects in multiple target tissues,
- The lack of standardized data to establish a quantitative relationship between a measurable genotoxic effect and the probability of experiencing an adverse health outcome,
- The lack of consensus on what increase of risk is societally deemed acceptable,
- The high variability of protective metabolic and DNA repair pathways,
- And, not least, the general particularities to be considered in connection with the risk assessment of genotoxic substances (e.g., default assumption of a non-threshold dose–response relationship, irreversibility, severity and time-delayed onset of health effects, combined effects on genome integrity).

For the time being, the opportunities of using genotoxicity data to support dose–response assessment remain to be evaluated case-by-case. The availability of appropriate information on the toxicokinetic profile, the genotoxic MoA and the quantitative relationships between genotoxic key events and the adverse health outcome being assessed might be considered a prerequisite. In any case, uncertainties arising from the restriction of current in vivo methods to specific endpoints and/or target tissues should be taken into account.

The use of genotoxicity dose–response data for prioritization purposes, e.g. in connection with the MOE approach, could be seen as a promising opportunity for routine application. Nevertheless, the routine use of genotoxicity data under the MOE approach should not be regarded as an option unless it can be demonstrated for a sufficiently large number of genotoxicants that the concordance between genotoxicity-based reference points and reference points based on downstream apical endpoints is generally high, or, should this not be the case, that observed differences are at least of a constant nature. This would allow to define a magnitude of the MOE that can be considered indicative of a low level of concern.

To further advance quantitative genotoxicity assessment, priority should be given to the development of new experimental methods to provide a deeper mechanistic understanding and a more comprehensive basis for the analysis of dose–response relationships. The latter will likely require the development of advanced genotoxicity markers that can be efficiently quantified in any relevant type of biological sample. These effect markers should be causally linked or at least well correlated with a specific adverse health outcome to increase the biological relevance of the identified reference points. In the long run, the integration of quantitative information on relevant key events along well-established AOPs should allow more informed assessment of the likely effect sizes at low, human relevant exposures and may also create new opportunities for the use of NAMs in connection with integrated approaches to genotoxicity testing and assessment.

## Supplementary Information

Below is the link to the electronic supplementary material.

Supplementary file1 (XLSX 24 KB)

## Data Availability

All data supporting the findings of this study are available within the paper and its Supplementary Information.

## Footnotes

1 In this document, the terms ‘reference point’ and ‘point of departure (PoD)’ are used synonymously.

2 The T25 is defined as “the chronic daily dose in mg per kg bodyweight which will give 25% of the animals tumours at a specific tissue site, after correction for spontaneous incidence, within the standard life span of that species” (Dybing et al. 1997).

3 The TD_50_ is defined as “the dose rate (in mg/kg body weight/day) that, if administered chronically for a standard period – the ‘standard lifespan’ of the species – will halve the mortality-corrected estimate of the probability of remaining tumorless throughout that period” (Peto et al. 1984).
